# Supplementary material for: Plant pathogenic bacterium can rapidly evolve tolerance to an antimicrobial plant allelochemical
Source: Evol Appl. 2022 Mar 18;15(5):735–50. doi: 10.1111/eva.13363 (PMC9108312; doi:10.1111/eva.13363)
Supplement: Supplementary file 1 — Fig S1 [file EVA-15-735-s002.docx]

**
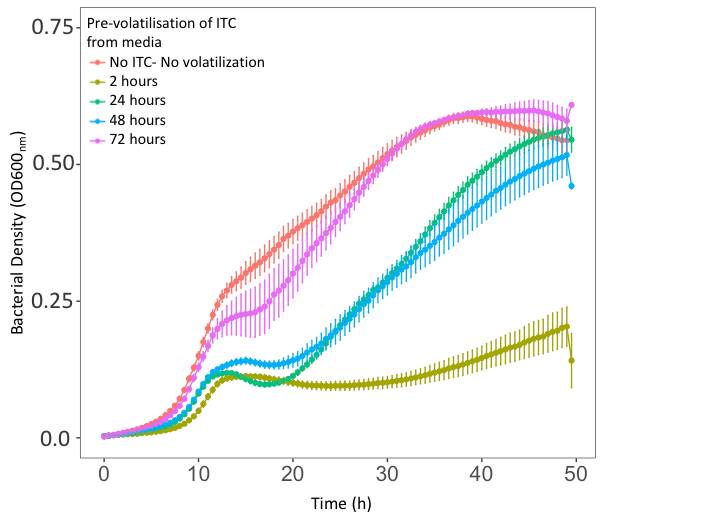
**

**Supplementary Figure 1.** **The effect of** **allyl-ITC pre-volatilisation for antibacterial activity against *R. solanacearum*.** *R. solanacearum* bacterial growth was measured in CPG media supplemented with 0 (No allyl-ITC) or 500 μM of allyl-ITC that had been allowed to volatilise for 2h, 24h, 48h or 72h before inoculation of bacterium (see key). All data points show the mean of eight technical replicates and bars show ±1 standard error of the mean (SEM).
