# Supplementary material for: Plant pathogenic bacterium can rapidly evolve tolerance to an antimicrobial plant allelochemical
Source: Evol Appl. 2022 Mar 18;15(5):735–50. doi: 10.1111/eva.13363 (PMC9108312; doi:10.1111/eva.13363)
Supplement: Supplementary file 2 — Fig S2 [file EVA-15-735-s004.docx]

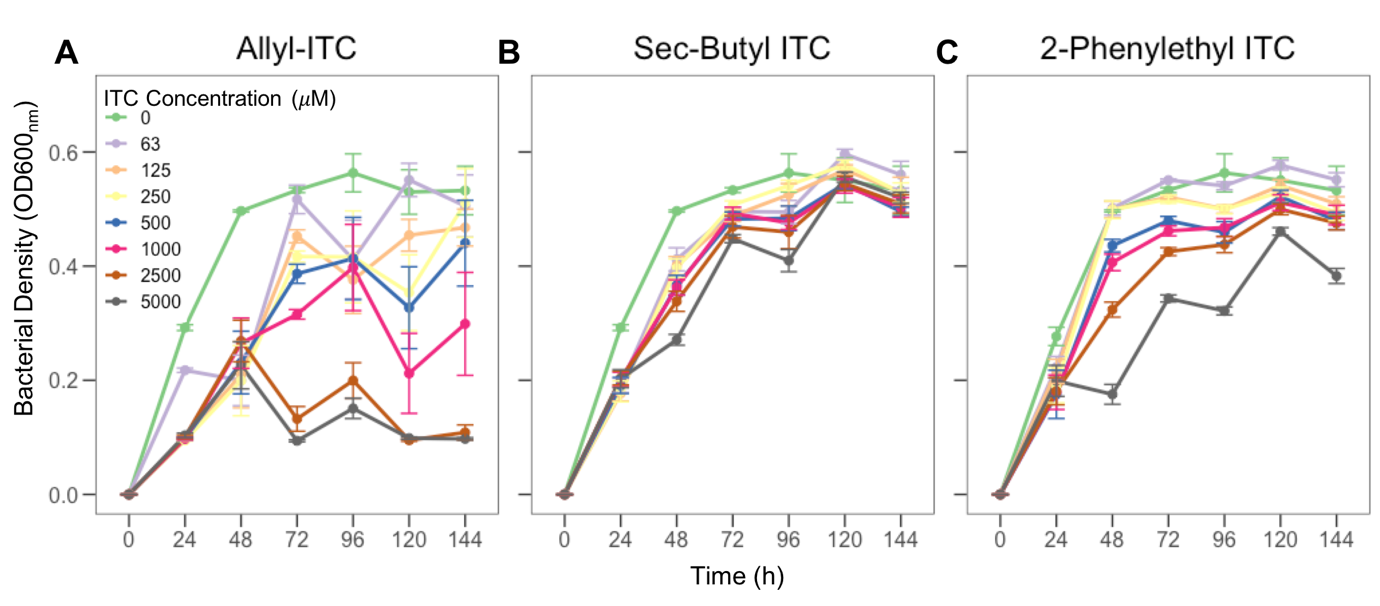


**Supplementary Figure 2.** **Effects of allyl, sec-butyl and 2-phenylethyl ITCs on *R. solanacearum* growth at different ITC concentrations.** In all panels, *R. solanacearum* bacterial densities are shown on the Y-axis as optical density (OD600_nm_), measured at 24-hour intervals (X-axis), and different line colours refer to different ITC concentrations (see key in A). All data points show the mean of eight technical replicates and bars show ±1 standard error of the mean (SEM).
