# Supplementary material for: Plant pathogenic bacterium can rapidly evolve tolerance to an antimicrobial plant allelochemical
Source: Evol Appl. 2022 Mar 18;15(5):735–50. doi: 10.1111/eva.13363 (PMC9108312; doi:10.1111/eva.13363)
Supplement: Supplementary file 3 — Fig S3 [file EVA-15-735-s007.docx]

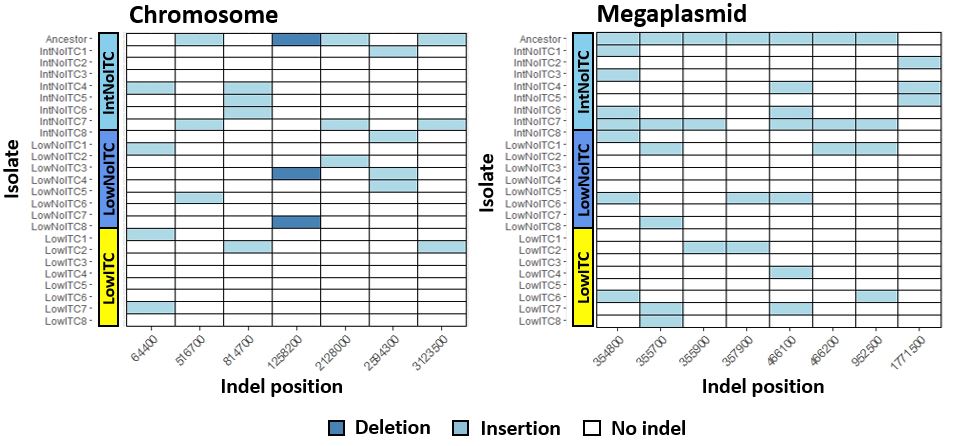


**Supplementary Figure 2. Presence and absence of intermediate indels found in more than two isolates in the chromosome and megaplasmid.** The X-axis shows the indel position rounded to the nearest 100bp. The Y-axis shows isolates grouped as shown in Figure 5.
