# Supplementary material for: Plant pathogenic bacterium can rapidly evolve tolerance to an antimicrobial plant allelochemical
Source: Evol Appl. 2022 Mar 18;15(5):735–50. doi: 10.1111/eva.13363 (PMC9108312; doi:10.1111/eva.13363)
Supplement: Supplementary file 4 — Fig S4 [file EVA-15-735-s005.docx]

**
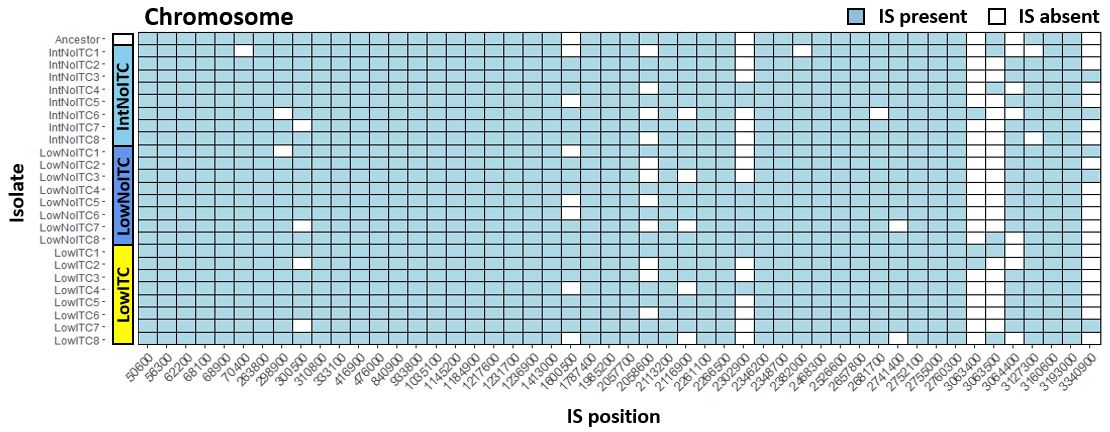
**

**
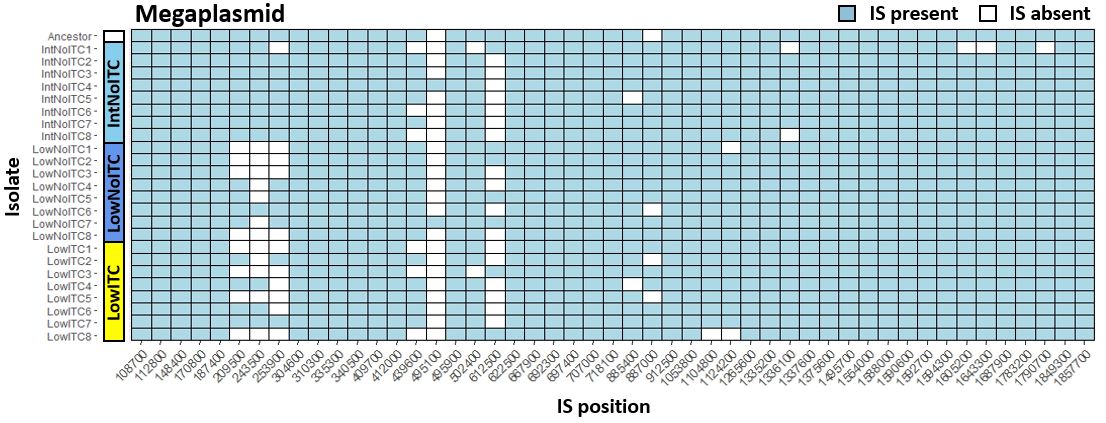
**

**Supplementary Figure 4. Presence and absence of insertion sequences in the chromosome and megaplasmid of *R. solanacearum*.** The X and Y-axes show the insertion sequence position and experimental isolate, respectively, as outlined in Figure 6.
