# Supplementary material for: Plant pathogenic bacterium can rapidly evolve tolerance to an antimicrobial plant allelochemical
Source: Evol Appl. 2022 Mar 18;15(5):735–50. doi: 10.1111/eva.13363 (PMC9108312; doi:10.1111/eva.13363)
Supplement: Supplementary file 5 — Table S1 [file EVA-15-735-s001.docx]

**Supplementary Table 1.** **The mean density reduction (%) of *R. solanacearum* bacterium when exposed to 500 µM or 1000 µM of allyl, sec-butyl and 2-phenylethyl ITCs in CPG growth media after 24h, 48h or 72h relative to when grown in the absence of ITCs**  based on the data presented in Supplementary Fig. 2.

| **ITC Type and Concentration (µM)** | **Time (h)** | **Bacterial density reduction (%) compared to control** |
| --- | --- | --- |
| Allyl-ITC, 500 | 24 | 66 |
|  | 48 | 54 |
|  | 72 | 27 |
| Allyl-ITC, 1000 | 24 | 66 |
|  | 48 | 47 |
|  | 72 | 41 |
| Sec-Butyl ITC, 500 | 24 | 33 |
|  | 48 | 26 |
|  | 72 | 9 |
| Sec-Butyl ITC, 1000 | 24 | 30 |
|  | 48 | 27 |
|  | 72 | 8 |
| 2-Phenylethyl ITC, 500 | 24 | 39 |
|  | 48 | 13 |
|  | 72 | 10 |
| 2-Phenylethyl ITC, 1000 | 24 | 38 |
|  | 48 | 18 |
|  | 72 | 13 |
