# Supplementary material for: Plant pathogenic bacterium can rapidly evolve tolerance to an antimicrobial plant allelochemical
Source: Evol Appl. 2022 Mar 18;15(5):735–50. doi: 10.1111/eva.13363 (PMC9108312; doi:10.1111/eva.13363)
Supplement: Supplementary file 6 — Table S2 [file EVA-15-735-s003.docx]

|  | **Doubling time (h)** | |  | **Estimate total number of generations per transfer frequency cycle** | |  | **Estimated number of generations experienced during the 16-day selection experiment** | |
| --- | --- | --- | --- | --- | --- | --- | --- | --- |
| **Growth period** | **No ITC** | **ITC** | **Transfer frequency** | **No ITC** | **ITC** | **Transfer frequency** | **No ITC** | **ITC** |
| 0-24h | 1.4 | 1.77 | High | 17.1 | 13.5 | High | 274.2 | 216.9 |
| 24-48h | 9.5 | 14.1 | Intermediate | 19.6 | 15.2 | Intermediate | 157.3 | 122 |
| 48-72h | 26.5 | 17.2 | Low | 20.5 | 16.6 | Low | 120 | 96.8 |

**Supplementary Table 2.** Estimation of ancestral *R. solanacearum* clone doubling time in the presence and absence of ITC during three growth periods of the transfer frequency cycles (0-24h, 24-48h and 48-72h). This information was used to estimate the total number of generations per transfer frequency cycle and the 16-day long selection experiment. The doubling time was calculated based on the ancestral *R. solanacearum* clone cell densities (CFU per mL) at 0h, 24h, 48h and 72h sampling time points in CPG media with and without allyl-ITC (500 μM).
