# Supplementary material for: Plant pathogenic bacterium can rapidly evolve tolerance to an antimicrobial plant allelochemical
Source: Evol Appl. 2022 Mar 18;15(5):735–50. doi: 10.1111/eva.13363 (PMC9108312; doi:10.1111/eva.13363)
Supplement: Supplementary file 7 — Table S3 [file EVA-15-735-s006.docx]

**Supplementary Table 3. Prophage information of ancestral and evolved *R. solanacearum* isolates with information on the left and right flanking genes determined uding the UY031 reference genome.** Replicates are named by treatments as follows, IntNoITC= Intermediate transfer frequency, no ITC; LowNoITC= Low transfer frequency, no ITC; LowITC= Low transfer frequency, ITC.

| **Clone** | **Prophage** | **Left flank UY031 position** | **Right flank UY031 position** | **Length (kb)** | **GC content (%)** | **Total proteins #** |
| --- | --- | --- | --- | --- | --- | --- |
| UY031 | Unclassified A | - | - | 37 | 62.76 | 44 |
|  | RS551 | - | - | 13.4 | 61.24 | 16 |
|  | PHAGE_Vibrio_VHML_NC_004456 | - | - | 18.5 | 64.64 | 29 |
| Ancestor | Unclassified A | NZ_CP012687.1:1121824-1126823 | NZ_CP012687.1:1162291-1162775 | 35.4 | 62.85 | 42 |
|  | RS551 | NZ_CP012687.1:1218220-1223219 | NZ_CP012687.1:1236384-1237639 | 13.1 | 58.58 | 17 |
| IntNoITC1 | Unclassified A | NZ_CP012687.1:1121824-1126823 | NZ_CP012687.1:1162291-1162775 | 35.4 | 62.85 | 42 |
|  | RS551 | NZ_CP012687.1:1218233-1223232 | NZ_CP012687.1:1236385-1237640 | 13.1 | 58.58 | 17 |
| IntNoITC2 | Unclassified A | NZ_CP012687.1:1121824-1126823 | NZ_CP012687.1:1162291-1162775 | 35.4 | 62.85 | 42 |
|  | RS551 | NZ_CP012687.1:1218233-1223232 | NZ_CP012687.1:1236385-1237640 | 13.1 | 58.58 | 17 |
| IntNoITC3 | Unclassified A | NZ_CP012687.1:1121823-1126822 | NZ_CP012687.1:1162290-1162775 | 35.4 | 62.85 | 43 |
|  | RS551 | NZ_CP012687.1:1218233-1223232 | NZ_CP012687.1:1236385-1237640 | 13.1 | 58.58 | 17 |
| IntNoITC4 | Unclassified A | NZ_CP012687.1:1121823-1126822 | NZ_CP012687.1:1162290-1162775 | 35.4 | 62.85 | 43 |
|  | RS551 | NZ_CP012687.1:1218233-1223232 | NZ_CP012687.1:1236385-1237640 | 13.1 | 58.58 | 18 |
| IntNoITC5 | Unclassified A | NZ_CP012687.1:1121824-1126823 | NZ_CP012687.1:1162291-1162775 | 35.4 | 62.85 | 42 |
|  | RS551 | NZ_CP012687.1:1218233-1223232 | NZ_CP012687.1:1236385-1237640 | 13.1 | 58.58 | 18 |
| IntNoITC6 | Unclassified A | NZ_CP012687.1:1121823-1126822 | NZ_CP012687.1:1162290-1162775 | 35.4 | 62.85 | 43 |
|  | RS551 | NZ_CP012687.1:1218233-1223232 | NZ_CP012687.1:1236385-1237640 | 13.1 | 58.58 | 18 |
| IntNoITC7 | Unclassified A | NZ_CP012687.1:1121824-1126823 | NZ_CP012687.1:1162291-1162775 | 35.4 | 62.85 | 43 |
|  | RS551 | NZ_CP012687.1:1218233-1223232 | NZ_CP012687.1:1218233-1223232 | 13.1 | 58.58 | 17 |
| IntNoITC8 | Unclassified A | NZ_CP012687.1:1121823-1126822 | NZ_CP012687.1:1162290-1162775 | 35.4 | 62.85 | 41 |
|  | RS551 | NZ_CP012687.1:1218233-1223232 | NZ_CP012687.1:1236385-1237640 | 13.1 | 58.58 | 17 |
| LowNoITC1 | Unclassified A | NZ_CP012687.1:1121824-1126823 | NZ_CP012687.1:1162291-1162775 | 35.4 | 62.85 | 42 |
|  | RS551 | NZ_CP012687.1:1218233-1223232 | NZ_CP012687.1:1236385-1237640 | 13.1 | 58.58 | 17 |
| LowNoITC2 | Unclassified A | NZ_CP012687.1:1121824-1126823 | NZ_CP012687.1:1162291-1162775 | 35.4 | 62.85 | 42 |
|  | RS551 | NZ_CP012687.1:1218233-1223232 | NZ_CP012687.1:1236385-1237640 | 13.1 | 58.58 | 18 |
| LowNoITC3 | Unclassified A | NZ_CP012687.1:1121823-1126822 | NZ_CP012687.1:1162290-1162775 | 35.4 | 62.85 | 41 |
|  | RS551 | NZ_CP012687.1:1218233-1223232 | NZ_CP012687.1:1236385-1237640 | 13.1 | 58.58 | 18 |
| LowNoITC4 | Unclassified A | NZ_CP012687.1:1121823-1126822 | NZ_CP012687.1:1162290-1162775 | 35.4 | 62.85 | 43 |
|  | RS551 | NZ_CP012687.1:1218233-1223232 | NZ_CP012687.1:1236385-1237640 | 13.1 | 58.58 | 17 |
| LowNoITC5 | Unclassified A | NZ_CP012687.1:1121823-1126822 | NZ_CP012687.1:1162290-1162775 | 35.4 | 62.85 | 43 |
|  | RS551 | NZ_CP012687.1:1218220-1223219 | NZ_CP012687.1:1236385-1237640 | 13.1 | 58.58 | 18 |
| LowNoITC6 | Unclassified A | NZ_CP012687.1:1121823-1126822 | NZ_CP012687.1:1162290-1162775 | 35.4 | 62.85 | 41 |
|  | RS551 | NZ_CP012687.1:1218220-1223219 | NZ_CP012687.1:1236384-1237640 | 13.1 | 58.58 | 18 |
| LowNoITC7 | Unclassified A | NZ_CP012687.1:1121823-1126822 | NZ_CP012687.1:1162290-1162775 | 35.4 | 62.85 | 41 |
|  | RS551 | NZ_CP012687.1:1218220-1223219 | NZ_CP012687.1:1236384-1237640 | 13.1 | 58.58 | 18 |
| LowNoITC8 | Unclassified A | NZ_CP012687.1:1121824-1126823 | NZ_CP012687.1:1162291-1162775 | 35.4 | 62.85 | 42 |
|  | RS551 | NZ_CP012687.1:1218220-1223219 | NZ_CP012687.1:1236384-1237640 | 13.1 | 58.58 | 17 |
| LowITC1 | Unclassified A | NZ_CP012687.1:1121824-1126823 | NZ_CP012687.1:1162291-1162775 | 35.4 | 62.85 | 43 |
|  | RS551 | NZ_CP012687.1:1218220-1223219 | NZ_CP012687.1:1236384-1237640 | 13.1 | 58.58 | 17 |
| LowITC2 | Unclassified A | NZ_CP012687.1:1121823-1126822 | NZ_CP012687.1:1162290-1162775 | 35.4 | 62.85 | 43 |
|  | RS551 | NZ_CP012687.1:1218220-1223219 | NZ_CP012687.1:1236384-1237640 | 13.1 | 58.58 | 17 |
| LowITC3 | Unclassified A | NZ_CP012687.1:1121824-1126823 | NZ_CP012687.1:1162291-1162775 | 35.4 | 62.85 | 42 |
|  | RS551 | NZ_CP012687.1:1218220-1223219 | NZ_CP012687.1:1236384-1237640 | 13.1 | 58.58 | 18 |
| LowITC4 | Unclassified A | NZ_CP012687.1:1121824-1126823 | NZ_CP012687.1:1162291-1162775 | 35.4 | 62.85 | 42 |
|  | RS551 | NZ_CP012687.1:1218220-1223219 | NZ_CP012687.1:1236384-1237639 | 13.1 | 58.58 | 17 |
| LowITC5 | Unclassified A | NZ_CP012687.1:1121823-1126822 | NZ_CP012687.1:1162290-1162775 | 35.4 | 62.85 | 41 |
|  | RS551 | NZ_CP012687.1:1218220-1223219 | NZ_CP012687.1:1236384-1237639 | 13.1 | 58.58 | 18 |
| LowITC6 | Unclassified A | NZ_CP012687.1:1121824-1126823 | NZ_CP012687.1:1162291-1162775 | 35.4 | 62.85 | 42 |
|  | RS551 | NZ_CP012687.1:1218220-1223219 | NZ_CP012687.1:1236384-1237639 | 13.1 | 58.58 | 17 |
| LowITC7 | Unclassified A | NZ_CP012687.1:1121823-1126822 | NZ_CP012687.1:1162290-1162775 | 35.4 | 62.85 | 41 |
|  | RS551 | NZ_CP012687.1:1218220-1223219 | NZ_CP012687.1:1236384-1237639 | 13.1 | 58.58 | 18 |
| LowITC8 | Unclassified A | NZ_CP012687.1:1121823-1126822 | NZ_CP012687.1:1162290-1162775 | 35.4 | 62.85 | 41 |
|  | RS551 | NZ_CP012687.1:1218220-1223219 | NZ_CP012687.1:1236384-1237639 | 13.1 | 58.58 | 18 |
